# Hispolon Methyl Ether, a Hispolon Analog, Suppresses the SRC/STAT3/Survivin Signaling Axis to Induce Cytotoxicity in Human Urinary Bladder Transitional Carcinoma Cell Lines

**DOI:** 10.3390/ijms24010138

**Published:** 2022-12-21

**Authors:** Min-Yung Kuo, Wei-Ting Yang, Yann-Jen Ho, Ge-Man Chang, Hsiung-Hao Chang, Chao-Yu Hsu, Chia-Che Chang, Yi-Hsin Chen

**Affiliations:** 1 Pediatric Surgery Division, Department of Surgery, Tungs’ Taichung MetroHarbor Hospital, Taichung 402202, Taiwan; 2 Institute of Biomedical Sciences, National Chung Hsing University, Taichung 402202, Taiwan; 3 Department of Life Sciences, National Chung Hsing University, Taichung 402202, Taiwan; 4 Division of Urology, Department of Surgery, Tungs’ Taichung MetroHarbor Hospital, Taichung 402202, Taiwan; 5 Ph.D. Program in Translational Medicine, Rong Hsing Research Center for Translational Medicine, The iEGG and Animal Biotechnology Research Center, National Chung Hsing University, Taichung 402202, Taiwan; 6 Department of Medical Laboratory Science and Biotechnology, Asia University, Taichung 413305, Taiwan; 7 Department of Medical Research, China Medical University Hospital, Taichung 404327, Taiwan; 8 Traditional Herbal Medicine Research Center, Taipei Medical University Hospital, Taipei 110301, Taiwan; 9 Department of Nephrology, Taichung Tzu Chi Hospital, Buddhist Tzu Chi Medical Foundation, Taichung 427213, Taiwan; 10 School of Medicine, Tzu Chi University, Hualein 970374, Taiwan

**Keywords:** hispolon methyl ether, STAT3, SRC, survivin, apoptosis, bladder cancer

## Abstract

Bladder cancer is a leading human malignancy worldwide. Signal transducer and activator of transcription (STAT) 3 is an oncogenic transcription factor commonly hyperactivated in most human cancers, including bladder cancer. Notably, preclinical evidence has validated STAT3 blockade as a promising therapeutic strategy for bladder cancer. Hispolon Methyl Ether (HME) is a structural analog of hispolon, an anticancer component of the medicinal mushroom *Phellinus linteus*. Thus far, HME’s anticancer activity and mechanisms remain largely unknown. We herein report HME was cytotoxic, more potent than cisplatin, and proapoptotic to various human bladder transitional carcinoma cell lines. Of note, HME blocked STAT3 activation, evidenced by HME-elicited reduction in tyrosine 705-phosphorylated STAT3 levels constitutively expressed or induced by interleukin-6. Significantly, HME-induced cytotoxicity was abrogated in cells expressing a dominant-active STAT3 mutant (STAT3-C), confirming STAT3 blockage as a pivotal mechanism of HME’s cytotoxic action. We further revealed that survivin was downregulated by HME, while its levels were rescued in STAT3-C-expressing cells. Moreover, survivin overexpression abolished HME-induced cytotoxicity, illustrating survivin as a central downstream mediator of STAT3 targeted by HME. Lastly, HME was shown to lower tyrosine 416-phosphorylated SRC levels, suggesting that HME inhibits STAT3 by repressing the activation of SRC, a STAT3 upstream kinase. In conclusion, we present the first evidence of HME’s anti-bladder cancer effect, likely proceeding by evoking apoptosis through suppression of the antiapoptotic SRC/STAT3/survivin signaling axis.

## 1. Introduction

Bladder cancer overwhelms over 430,000 people worldwide per year and remains a significant malignancy globally [1]. The risk factors of bladder cancer include being male, elderly, smoking, and particular carcinogens such as benzene chemicals and aromatic amines [2]. In total, 70% of bladder cancer are non-muscle-invasive types, with 5-year recurrent free survival of 43% in a low-risk group, but 21% with high risk will progress to muscle-invasive bladder cancer [3]. However, the prognosis is poor if metastasis develops, with a median survival of 13 to 15 months [4]. Despite the era of novel therapy in other cancers, there has been no treatment progress for bladder cancer in these years. Transurethral resection of bladder tumors with intravesical chemotherapy in the operation room is given to patients with nonmuscle invasive bladder cancer [5]. Systemic treatment with platinum-based chemotherapy, such as cisplatin, is essential for reducing recurrence, especially for muscle-invasive bladder cancer after an operation [6]. However, the side effect of chemotherapy, including myelosuppression, nephrotoxicity, and gastrointestinal upset, still hurdles the therapy. Thus, finding a novel effective treatment is urgent for this common malignancy.

Signal transducer and activator of transcription (STAT) 3 is a member of the STAT family of transcription factors. Physiologically, STAT3 regulates immunity along with cell proliferation, survival, and apoptosis [7]. Under normal conditions, STAT3 remains latent in the cytosol until activation by stimulation with cytokines such as interleukin-6 (IL-6) or growth factors through the IL-6 receptor (IL-6R) and receptor tyrosine kinases (RTKs), respectively. Activation of IL-6R and RTKs promotes the phosphorylation/activation of Janus kinases (JAKs) or nonreceptor tyrosine kinase SRC (through RTKs) to phosphorylate STAT3 at the tyrosine 705 residue, leading to the dimerization and ensuing nuclear translocation of STAT3 for transactivation of STAT3 target genes [8,9,10]. Normally a transient and tightly controlled event, STAT3 activation is nevertheless deregulated and remains constitutive in malignant cells. Aberrant STAT3 hyperactivation is present in most human cancers and is integral to provoking malignant phenotypes [11]. In bladder cancer, persistent STAT3 activation is proven to sustain cell proliferation and survival, facilitate metastasis, and promote chemoresistance [12]. Recent evidence further underpins the STAT3 signaling pathway as a central mechanism whereby oncogenes promote bladder cancer progression [13,14,15,16,17]. It is also noteworthy that pharmacological inhibition of STAT3 has been demonstrated to effectively suppress bladder cancer cell progression in vitro and in vivo, thus validating STAT3 blockage as a potential strategy for bladder cancer therapy [18,19,20,21].

Hispolon (6-(3,4-dihydroxyphenyl)-4-hydroxyhexa-3,5-dien-2-one; C_12_H_12_O_4_) is a natural polyphenolic compound widely present in *Phellinus* genus mushrooms, including the traditional medicinal mushroom *Phellinus linteus* commonly used in East Asia [22]. Hispolon is a main bioactive component responsible for numerous health-beneficial activities of *P. linteus*, including antidiabetic, antiinflammatory, antioxidant, and anticancer [23,24,25]. Of note, hispolon has been demonstrated to induce in vitro cytotoxicity against a broad range of human cancer cell lines and retard xenograft tumor growth of certain types of human cancers [24,25], making it a potential lead compound for developing novel anticancer agents. Accordingly, a number of structurally modified analogs of hispolon have been generated to evaluate their anticancer potentials [26,27]. In this regard, we have reported that dehydroxyhispolon methyl ether (DHME), one of the hispolon analogs, selectively induces cytotoxicity against a panel of human colorectal cancer (CRC) cell lines and further revealed DHME’s ant-CRC effect is exerted by evoking apoptosis through sabotaging the WNT/β-catenin signaling axis, a well-defined oncogenic pathway of CRC [28]. On the other hand, the anticancer effect and underlying mechanisms elicited by other hispolon analogs, such as hispolon methyl ether (HME), remain unidentified and thus warrant further investigation.

Herein, we present the first report demonstrating HME’s anti-bladder cancer effect and its mechanisms of anticancer action. In brief, we validated HME’s cytotoxic effect, more potent than that of cisplatin, on a panel of human bladder cancer cell lines. This cytotoxic action is attributed to the induction of bladder cancer cell apoptosis by thwarting the antiapoptotic SRC/STAT3/survivin signaling axis. Our discovery thereby implicates HME as a potential agent for bladder cancer chemotherapy.

## 2. Results

### 2.1. HME Is Cytotoxic to a Panel of Human Urinary Bladder TCC Cell Lines

**Figure 1:**
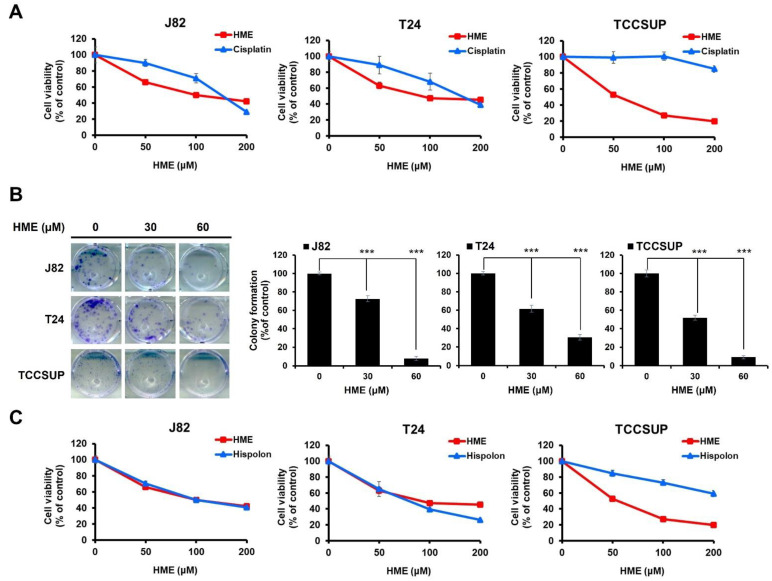
HME’s cytotoxic effect on human bladder TCC cell lines. (**A**) HME induces stronger cytotoxicity than cisplatin against human bladder TCC cells. Human bladder TCC cell lines J82, T24, and TCCSUP were treated with HME or cisplatin (0~200 μM) for 48 h, followed by cell viability determination using MTS assay. (**B**) HME impairs the colony-forming capacity of human bladder TCC cells. The levels of clonogenicity of HME-treated J82, T24, and TCCSUP cells were determined as described in Section 4. ***: *p* < 0.001. (**C**) Higher cytotoxic effect of HME than hispolon on TCCSUP cells while comparable cytotoxicity of these two drugs against J82 and T24 cells. J82, T24, and TCCSUP were treated with HME or hispolon (0~200 μM) for 48 h, followed by cell viability determination using MTS assay.

To evaluate HME’s potential as an anti-bladder cancer agent, we began by testing whether HME induces cytotoxicity against human bladder cancer cells using an MTS assay. Cisplatin, one of the first-line chemotherapeutic agents for bladder cancer treatment [4], was included as a positive control for bladder cancer cytotoxicity. We observed that HME dose-dependently reduced the viability of diverse human urinary bladder transitional cell carcinoma (TCC) cell lines, including J82, T24, and TCCSUP, and HME’s cytotoxic potency was stronger than cisplatin (Figure 1A). Furthermore, HME suppressed the clonogenicity of these bladder TCC cell lines (Figure 1B). These results demonstrated that HME is cytotoxic against human bladder cancer cells. We also examined the cytotoxic effect of HME and its parental molecule, hispolon, on the above bladder TCC cell lines. As shown in Figure 1C, HME displayed stronger cytotoxicity than hispolon against TCCSUP cells while showing comparable cytotoxicity with hispolon to J82 and T24 cells.

### 2.2. HME-Induced Bladder Cancer Cytotoxicity Involves Apoptosis Induction

**Figure 2:**
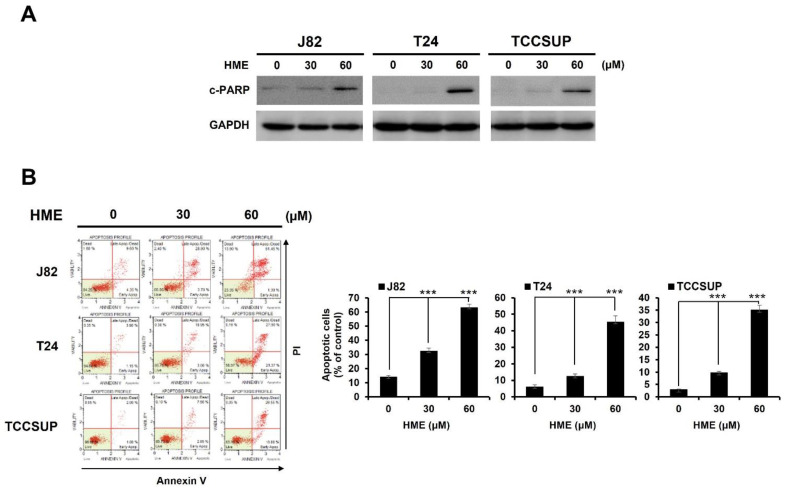
HME’s proapoptotic effect on human bladder TCC cell lines. (**A**) Induction of PARP cleavage by HME. J82, T24, and TCCSUP cells were treated with HME (0, 30, 60 μM) for 24 h, followed by immunoblotting to evaluate the levels of PARP cleavage. The levels of glyceraldehyde-3-phosphate dehydrogenase (GAPDH) were used as a control for equal loading. c-PARP: cleaved PARP. (**B**) Increase of annexin V-positive (apoptotic) cell population by HME. J82, T24, and TCCSUP cells were treated with HME (0, 30, 60 μM) for 24 h, followed by flow cytometry-based annexin V/propidium iodide (PI) dual staining as described in Section 4. ***: *p* < 0.001.

The nature of HME-induced cytotoxicity was next explored. Immunoblot analysis indicated that HME treatment led to an increase in the levels of cleaved poly-ADP-ribose polymerase (PARP) (c-PARP) in all three bladder TCC cell lines, illustrating that HME elicited caspases activation and hence the induction of apoptosis (Figure 2A). Further flow cytometry analysis uncovered an obvious elevation of annexin V-positive (i.e., apoptotic) populations in HME-exposed cells, confirming HME’s proapoptotic action (Figure 2B). Thus, these data argued that HME evokes bladder cancer cytotoxicity likely by inducing apoptotic cell death.

### 2.3. STAT3 Blockage Is Required for HME-Induced Bladder Cancer Cytotoxicity

**Figure 3:**
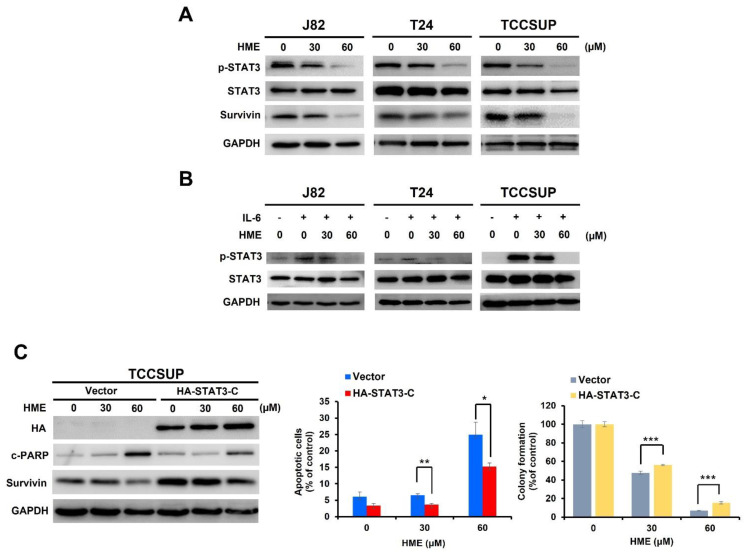
Blockade of STAT3 activation is a vital mechanism of HME’s anti-bladder cancer action. (**A**) HME inhibits constitutive STAT3 activation in human bladder TCC cell lines. J82, T24, and TCCSUP cells were treated with HME (0, 30, 60 μM) for 24 h, followed by immunoblotting for the levels of tyrosine 705-phosphorylated STAT3 (p-STAT3), total STAT3, and survivin, a canonical STAT3 target gene. GAPDH levels were used for equal loading control. (**B**) HME inhibits IL-6-induced STAT3 activation in human bladder TCC cell lines. J82, T24, and TCCSUP cells were treated without or with HME (0, 30, 60 μM) for 24 h in the absence or presence of IL-6 (100 ng/mL) stimulation for 30 min. The lysates from each treatment were subjected to immunoblotting for the levels of p-STAT3 and total STAT3. GAPDH levels were used for controlling equal loading. (**C**) Ectopic expression of STAT3-C (a dominant-active STAT3 mutant) impairs HME’s anti-bladder cancer activity. TCCSUP cells with stable expression of HA-tagged STAT3-C (HA-STAT3-C) and the respective vector control were treated with HME (0, 30, 60 μM) for 24 h, followed by immunoblotting to determine the levels of HA, c-PARP, and survivin. GAPDH levels were used for equal loading control (Left). Moreover, the HA-STAT3 stable clones and the respective vector control clones after 24 h-treatment with HME (0, 30, 60 μM) were subjected to annexin V/PI dual staining assay (center) and clonogenicity (right) for the evaluation of HME-induced apoptosis and cytotoxicity, respectively. *: *p* < 0.05; **: *p* < 0.01; ***: *p* < 0.001.

Accumulating evidence has underscored the link between aberrant STAT3 activation and the malignant progression of diverse human cancers, including bladder cancer [12]. Notably, we found HME suppressed the constitutive STAT3 activation in J82, TCCSUP, and T24 cells, as evidenced by the reduced levels of tyrosine 705-phosphorylated STAT3 (p-STAT3 (Y705)) along with the decreased expression of survivin, an STAT3 downstream target [29,30] (Figure 3A). Besides blocking constitutive STAT3 activation, we asked whether HME impairs IL-6-induced STAT3 activation in these bladder TCC cells. It is noteworthy that in all three cell lines, IL-6 alone stimulated a robust increase in p-STAT3 (Y705) levels, whereas IL-6-induced p-STAT3 (Y705) increase was abrogated when co-treated with HME (Figure 3B). Our findings revealed that HME inhibits both constitutive and IL-6-induced STAT3 activation in human bladder cancer cells, supporting HME as an inhibitor of STAT3 activation.

Given that HME suppresses STAT3 activation, we aimed to define the role of STAT3 inhibition in HME-mediated bladder cancer cell death. To address this, we generated a stable TCCSUP cell clone that expresses a dominant-active STAT3 mutant (STAT3-C) to withstand HME-mediated blockage of STAT3 activation (Figure S1). It is noteworthy that for this STAT3-C stable clone, both HME-triggered apoptosis induction and clonogenicity inhibition were clearly impaired, indicating that HME-induced bladder cancer cytotoxicity was sabotaged when HME fails to impede STAT3 activation (Figure 3C). Accordingly, these findings illustrate that blockade of STAT3 activation is required for HME-elicited cytotoxicity in bladder cancer cells.

### 2.4. HME Downregulates Survivin, a Canonical STAT3 Downstream Target, to Induce Bladder Cancer Cytotoxicity

**Figure 4:**
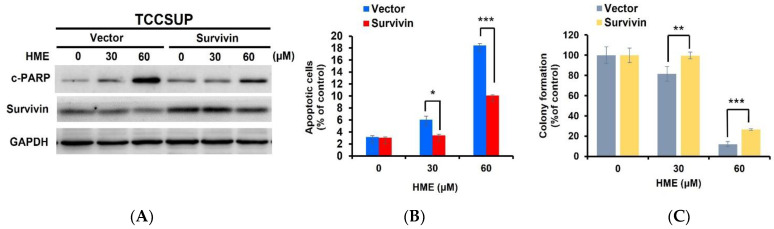
Ectopic survivin expression blunts HME-induced cytotoxic effects, confirming survivin downregulation as a pivotal mechanism of HME’s anti-bladder cancer action. TCCSUP stable clones of survivin and the respective vector controls were subjected to 24 h-treatment with HME (0, 30, 60 μM) and then subjected to immunoblotting for c-PARP, survivin, and GAPDH (loading control) (**A**), Annexin V/PI dual staining (**B**), and clonogenicity assay (**C**). *: *p* < 0.05; **: *p* < 0.01; ***: *p* < 0.001.

After establishing a pivotal role of STAT3 blockage in HME’s cytotoxic action, we further explored the candidate effectors downstream of STAT3 to mediate HME-induced bladder cancer cytotoxicity. Along this line, we found earlier that HME downregulates survivin, a vital pro-survival protein known as a STAT3 downstream target (Figure 3A). Indeed, ectopic STAT3-C expression rescued survivin from HME-induced downregulation (Figure 4A), confirming that HME lowers survivin levels by blocking STAT3 activation. We further asked whether survivin downregulation mediates HME-induced bladder cancer cytotoxicity. Of note, TCCSUP cells stably expressing survivin were refractory to HME-evoked induction of apoptosis and inhibition of clonogenicity (Figure 4B). Overall, we argue that HME blocks STAT3 activation to downregulate survivin, in turn promoting bladder cancer cell death.

### 2.5. HME Represses SRC to Block STAT3 Activation

**Figure 5:**
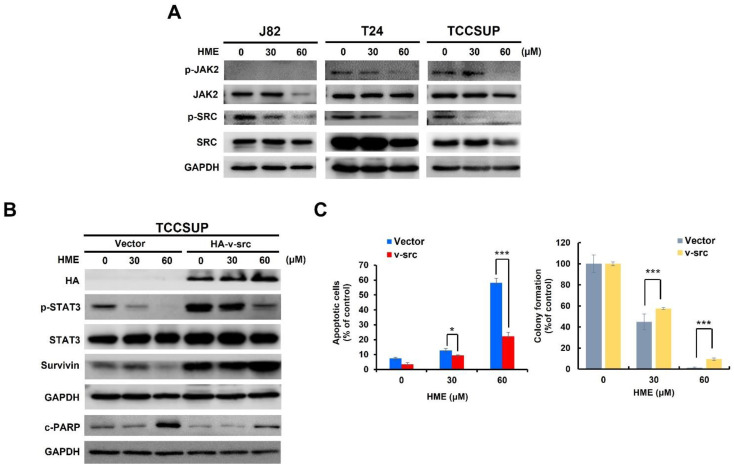
HME inhibits SRC to suppress the STAT3/survivin signaling axis. (**A**) HME inhibits SRC activation in all human bladder TCC cell lines examined. J82, T24, and TCCSUP cells were treated with HME (0, 30, 60 μM) for 24 h, followed by immunoblotting for the levels of tyrosine 1007/1008-phosphorylated JAK2 (p-JAK2), total JAK2, tyrosine 416-phosphorylated SRC (p-SRC), total SRC, and GAPDH (loading control). (**B**) Ectopic expression of v-src, a dominant-active SRC, rescues both p-STAT3 and survivin levels from HME-mediated suppression but also attenuates HME-induced PARP cleavage. TCCSUP stable clones of HA-v-src and the respective vector control were treated with HME (0, 30, 60 μM) for 24 h, followed by immunoblotting to evaluate the levels of HA, p-STAT3, total STAT3, survivin, c-PARP, and GAPDH (loading control). (**C**) v-src overexpression antagonizes the proapoptotic and cytotoxic effect of HME. TCCSUP stable clones of HA-v-src and the respective vector control were treated with HME (0, 30, 60 μM) for 24 h, followed by annexin V/PI dual staining assay (left) and clonogenicity assay (right) to examine the HME-induced apoptosis and cytotoxicity, respectively. *: *p* < 0.05; ***: *p* < 0.001.

How HME suppresses STAT3 activation in bladder cancer cells was next addressed. To this end, we began by testing the effect of HME on the activation status of JAK2 and SRC, two eminent STAT3 upstream kinases that phosphorylate STAT3′s tyrosine 705 to induce STAT3 activation [10]. Immunoblotting indicated that in both TCCSUP and T24 cells, HME reduced the levels of activated JAK2 (revealed by dual phosphorylation at JAK2′s tyrosine residues 1007 and 1008 (p-JAK2 (Y1007/1008)) while detecting no p-JAK2 (Y1007/1008) signals in the J82 cells (Figure 5A, upper two panels). In contrast, HME downregulated tyrosine 416-phosphorylated SRC (p-SRC (Y416)) in the J82, TCCSUP, and T24 cells dose-dependently (Figure 5A, lower two panels), arguing that the blockade of SRC activation is a general cellular event provoked by HME.

Given that SRC activation was inhibited in all HME-treated bladder cancer cell lines, we figured SRC is the primary target of HME for blocking STAT3 activation, in turn promoting bladder cancer cytotoxicity. To address this, we observed that HME failed to downregulate both p-STAT3 (Y705) and survivin nor upregulate c-PARP in TCCSUP cells stably expressing v-src, a dominant-active SRC mutant (Figure 5B). As a result, HME’s capacity to induce apoptosis and impede clonogenicity was crippled when SRC remained active (Figure 5C). Collectively, these data supported that HME blocks STAT3 activation by suppressing SRC activity in bladder cancer cells.

## 3. Discussion

In this study, we established the cytotoxic effect of HME, an analog of hispolon, on human bladder cancer cells and the underlying mechanism of action. Specifically, we demonstrated that HME is cytotoxic and proapoptotic to a panel of human bladder TCC cell lines and exhibits more potent cytotoxicity than Cisplatin (Figure 1 and Figure 2). Furthermore, HME was proved as a blocker for STAT3 activation, evidenced by the findings that HME inhibits both constitutive and IL-6-induced activation of STAT3, and STAT3 blockage is needed for HME to suppress the viability and clonogenicity of bladder cancer cells (Figure 3). Moreover, we showed that survivin downregulation by HME due to STAT3 blockage contributes to HME-induced bladder cancer cytotoxicity (Figure 4). Lastly, we verified that HME blocks STAT3 activation apparently via thwarting SRC activation (Figure 5). Altogether, the evidence supports that HME represses the SRC/STAT3/survivin signaling axis to inhibit cell viability and clonogenicity of bladder cancer cells, consequently exerting cytotoxicity. To the best of our knowledge, these findings of HME-induced cytotoxic effect on human bladder cancer cells and the underlying mechanisms of cytotoxic action have never been reported previously.

Recent studies have demonstrated that hispolon, a main pharmacological constituent of *P. linteus*, exerts a marked anticancer effect on diverse human cancer cell lines, including bladder cancer [24,25]. This notion prompted the exploration of chemically modified analogs of hispolon for better application as anticancer agents [26,27]. Along this line, we formerly identified DHME, one of the hispolon analogs, as a potent proapoptotic drug for a panel of CRC cell lines by targeting the WNT/β-catenin signaling, a well-known oncogenic pathway driving CRC genesis and progression [28]. In this study, we focused on HME, another hispolon analog, and unraveled the HME-mediated anti-bladder cancer activity along with the underlying mechanisms of action. Of note, Lu et al. previously reported that hispolon induces antiproliferation of T24 cells by arresting cell-cycle progression at the G2/M phase, likely through upregulating p21^CIP1^ as a result of ERK-mediated downregulation of MDM2 [31]. In view of that, it would be interesting to examine, besides its cytotoxic action, whether HME provokes antiproliferation of human bladder cancer cells through the similar mechanisms of action employed by Hispolon.

Mounting evidence has substantiated the notion that blockage of STAT3 activation is a promising therapeutic strategy for multiple human malignancies, including bladder cancer [12,18,19,20,21]. Notably, the present study first identified HME as a potent blocker for STAT3 signaling in bladder cancer cells, as HME markedly inhibited both constitutive and IL-6-inducible STAT3 activation (Figure 3A,B). Moreover, the crucial role of STAT3 blockage in mediating HME’s anti-bladder cancer action was confirmed by the abrogation of HME-elicited cytotoxicity when HME failed to mitigate STAT3 activation (Figure 3C,D). Thus, our discovery that HME is an inhibitor of STAT3 signaling implicates the translation potential of HME as a chemotherapeutic agent for bladder cancer. It is also noteworthy that numerous STAT3 inhibitors are actually natural products or natural product derivatives [32]. Our present finding thus adds HME as a new member of the growing list of natural product-based STAT3 inhibitors.

Data presented here underscored that survivin downregulation due to STAT3 blockage by HME is a vital mechanism of HME’s cytotoxic action on bladder cancer cells (Figure 4). Survivin is a member of the inhibitors of apoptosis (IAP) family of proteins and is known to promote multiple malignant features, including cancer cell proliferation, survival, drug resistance, and maintenance of cancer stem cells [33]. Survivin is also unique by its differential expression in tumors while barely expressed in normal tissues, making it a potential prognosis biomarker and a valuable target for cancer therapeutics [34,35]. For bladder cancer, survivin expression level has been shown to be positively associated with tumor grades, disease progression and recurrence, and mortality [36,37,38,39,40]. It is also noteworthy that preclinical studies have validated survivin downregulation as a promising therapeutic strategy for bladder cancer [41]. Altogether, these lines of evidence further underpin the translation potential of HME for bladder cancer therapy.

Our inquiry about how HME suppresses STAT3 activation revealed that SRC appears as the primary STAT3 upstream kinase targeted by HME, as the levels of tyrosine 416-phosphorylated SRC were markedly lowered in all HME-treated bladder cancer cell lines (Figure 5). In this context, HME likely suppresses STAT3 activation via impeding SRC-mediated STAT3 phosphorylation at tyrosine 705. Still, the question of how HME obstructs SRC activation remains unknown. Previous studies have established that stimulation with growth factors or cytokines such as IL-6 can trigger SRC activation [42,43], coinciding with our finding that HME impairs both constitutive and IL-6-induced activation of STAT3 (Figure 3A,B). Along this line, it is plausible to speculate that HME might block SRC activation directly or through interfering with the activation of receptor tyrosine kinases or the IL-6 receptor. It is also worth noting that our discovery about SRC inhibition by HME implies that, besides STAT3, additional signaling pathways downstream of SRC, such as AKT and MAPK [44,45,46,47,48], are likely involved in HME’s anticancer mechanisms of action in the context of bladder cancer. This question is warranted to be addressed in the follow-up studies.

**Figure 6:**
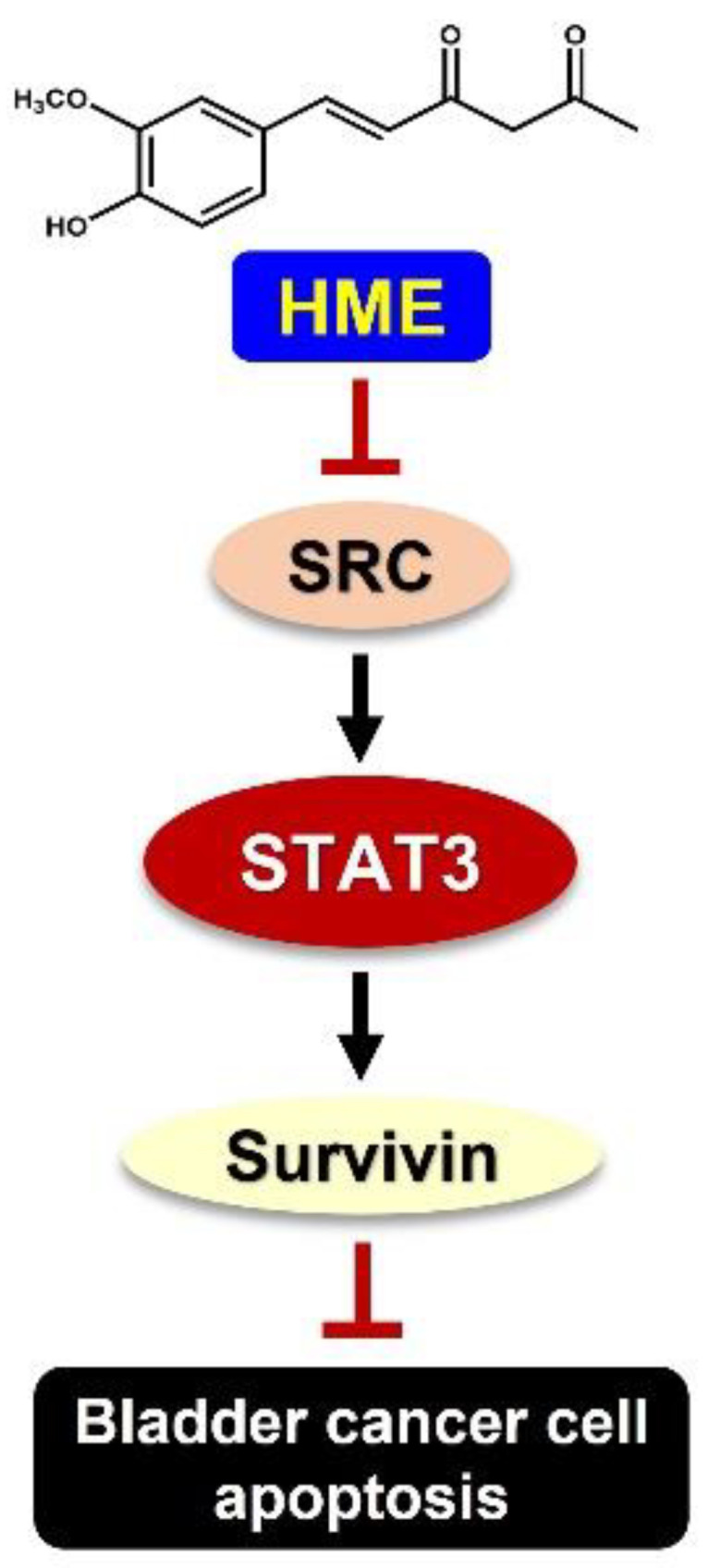
Schematic model of hispolon methyl ether (HME)-elicited anti-bladder cancer effect and the underlying mechanism of action based on the findings of this study. Briefly, HME induces cytotoxicity against bladder cancer cells by inducing apoptotic cell death by suppressing the antiapoptotic SRC/STAT3/survivin signaling pathway.

In conclusion, we first report HME’s cytotoxic effect on human bladder cancer cells via suppression of the antiapoptotic SRC/STAT3/survivin signaling axis (Figure 6). Considering the central role of this signaling pathway in bladder cancer development and progression, HME holds great potential as a chemotherapeutic agent for bladder cancer treatment.

## 4. Materials and Methods

### 4.1. Chemicals

Hispolon methyl ether was chemically synthesized according to the published procedure [27]. Hispolon was purchased from Enzo Life Sciences (Farmingdale, NY, USA). Cisplatin was obtained from AdooQ^®^ Bioscience (Irvine, CA, USA). Recombinant human IL-6 was acquired from PeproTech (Rehovot, ISR).

### 4.2. Plasmids

pBabe-HA-STAT3-C, the pBabe plasmid-based expression vector for ectopic expression of the dominant-active STAT3 mutant (STAT3 (A661C/N663C); STAT3-C) tagged with an N-terminal hemagglutinin (HA) epitope, has been described previously [49]. To construct the expression vector of survivin (pBabe-survivin), the open reading frame (ORF) of human *BIRC5* (Genebank number: NM_001168) was PCR-amplified from the cDNA pool of human colorectal cancer cell line HCT 116 (ATCC CCL-247™) using the following primer pair: 5′-GCCATTAACCGCCAGATTTG-3′ (forward) and 5′-CTAAGACATTGCTAAGGGGC-3′ (reverse). The PCR-amplified *BIRC5* ORF was TA-cloned into the pGEM-Teasy vector (Promega; Madison, WI, USA) for sequence verification. The sequence-verified BIRC5 ORF was then retrieved by EcoR I digestion and subcloned to the pBabe.puro vector at the EcoR I site, followed by validation of in-frame orientation. For generating pBabe-HA-v-src, the v-src-overexpressing vector, the v-src ORF was PCR-amplified using pLNCX chick src Y527F (Addgene plasmid #13660) as the template by the following primer pair: 5′-ACCGGTGGGAGCAGCAAGAGCAAG-3′ (forward) and 5′-CTATAGGTTCTCTCCAGGCTG-3′ (reverse). Then, the PCR-amplified product was TA-cloned, sequence verified, digested by Age I and Eco RI, and then directionally subcloned to the pBabe-HA vector established in our laboratory [49]. pLNCX chick src Y527F was a gift from Joan Brugge (Addgene plasmid #13660; http://n2t.net/addgene:13660; RRID:Addgene_13660 accessed on 28 November 2016).

### 4.3. Cell Culture

Human urinary bladder transitional cell carcinoma cell lines J82 (ATCC HTB-1™) and TCCSUP (ATCC HTB-5™) were both cultured in Eagle’s Minimum Essential Medium, while the T24 cell line (ATCC HTB-4™) in McCoy’s 5a medium in accordance with the recommendation of the American Type Culture Collection (ATCC) (Manassas, VA, USA). Cells were grown at 37 °C in a humidified environment with 5% CO_2_ in their respective culture media supplemented with non-essential amino acids, 1 mM sodium pyruvate, 10% fetal bovine serum, and 1% penicillin–streptomycin. All chemicals involved in the cell culture were purchased from Gibco Life Technologies (Carlsbad, CA, USA).

### 4.4. Cytotoxicity Assay

Short-term in vitro cytotoxicity was determined by the effect of drugs on cell viability using the CellTiter 96^®^ AQueous One Solution Cell Proliferation Assay (MTS) assay in accordance with the manufacturer’s protocol (Promega; Madison, WI, USA). Human bladder TCC cells were grown in 96-well culture plates (7 × 10^3^ cells/well) for 24 h before drug treatment, and the cell viability after drug treatment for 24 h and 48 h was measured thereafter. Long-term in vitro cytotoxicity was assessed by the effect of drugs on cells’ ability to form colonies (clonogenicity) after culture for 10~14 days. Clonogenicity assay was performed as described previously [49,50].

### 4.5. Apoptosis Assay

HME-induced human bladder TCC cell apoptosis was scored quantitatively using the Muse^®^ Annexin V & Dead Cell Assay Kit (Millipore; Burlington, MA, USA) as previously reported [49,50]. In short, 3 × 10^5^ cells grown on each well of a 6-well plate were subjected to HME treatment (0, 30, 60 μM) for 24 h, followed by resuspension using trypsinization, two-times washed with phosphate-buffered saline (PBS), and 20 min-incubation with 100 μL of Annexin V & Dead Cell reagent at room temperature in the dark. Thereafter, the annexin V-positive (apoptotic) cell population levels were gauged using flow cytometry analysis on the Muse^®^ Cell Analyzer (Millipore; Burlington, MA, USA).

### 4.6. Stable Clone Generation

TCCSUP cell clones stably expressing HA-STAT3-C, survivin, or HA-v-src were established by infecting TCCSUP cells with pBabe vector-derived retroviral particles carrying the ORFs of HA-STAT3-C, survivin, or v-src, followed by positive selection for 2 days with puromycin for the cells with successful infection. The puromycin-selected cell clones were then subjected to immunoblotting to confirm the ectopic expression of HA-STAT3, survivin, or HA-v-src proteins. The pBabe vector-derived retroviral particles were produced by following the established protocols reported previously [49,50].

### 4.7. Immunoblottinghme

Immunoblot analysis was conducted in accordance with our established procedures [49,50]. Primary antibodies against cleaved PARP (#9541), HA-tag (#3724), phospho-STAT3 (Tyr 705) (#9145), phospho-JAK2 (Tyr 1007/1008) (#3776), JAK2 (#3230), phospho-Src (Tyr 416) (#6743), and Src (#2108) were purchased from Cell Signaling Technology (Boston, MA, USA). Anti-Bcl-2 (GTX100064), anti-GAPDH (GTX110118), and anti-STAT3 (GTX104616) antibodies were obtained from GeneTex (Irvine, CA, USA). Anti-survivin antibody (10508-1-AP) was bought from Proteintech (Rosemont, IL, USA). All secondary antibodies were acquired from Jackson ImmunoResearch Laboratories (West Grove, PA, USA).

## Data Availability

Data will be available by corresponding author (chia_che@dragon.nchu.edu.tw) upon reasonable request.

## Supplementary Materials

The following supporting information can be downloaded at: https://www.mdpi.com/article/10.3390/ijms24010138/s1.
