# Supplementary material for: Hispolon Methyl Ether, a Hispolon Analog, Suppresses the SRC/STAT3/Survivin Signaling Axis to Induce Cytotoxicity in Human Urinary Bladder Transitional Carcinoma Cell Lines
Source: Int J Mol Sci. 2022 Dec 21;24(1):138. doi: 10.3390/ijms24010138 (PMC9820424; doi:10.3390/ijms24010138)
Supplement: Supplementary file 1 [file ijms-24-00138-s001.zip › ijms-2088333-supplementary.pdf]

Supplementary materials for ijms-2088333

Hispolon Methyl Ether, A Hispolon Analog, Suppresses the SRC/STAT3/Survivin Signaling Axis to Induce Cytotoxicity in Human Urinary Bladder Transitional Carcinoma Cell Lines

Min-Yung Kuo [1,2], Wei-Ting Yang [3], Yann-Jen Ho [3], Ge-Man Chang [2], Hsiung-Hao Chang [3], Chao-Yu Hsu [4,5], Chia-Che Chang [2,3,5,6,7,8,*] and Yi-Hsin Chen [9,10,*]

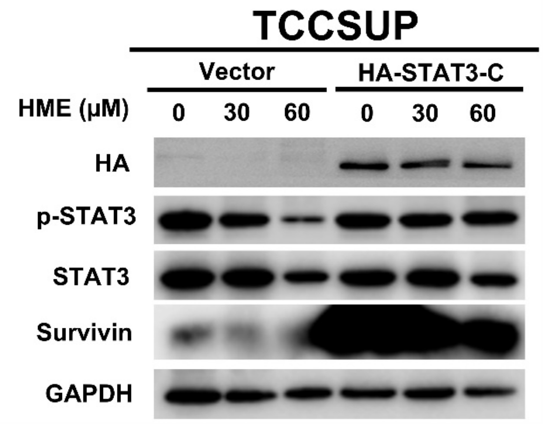

Figure S1. Ectopic expression of HA-STAT3-C sustains STAT3 activity following HME treatment. TCCSUP cell clones stably expressing HA-STAT3-C or the corresponding vector control were subjected to 24 h-treatment with HME (0, 30, 60 μM), followed by immunoblotting for the levels of HA, tyrosine 705-phosphorylated STAT3 (p-STAT3), total STAT3, Survivin, and GAPDH (loading control). The constant levels of p-STAT3 and Survivin in HME-treated STAT3-C stable clones illustrate the resistance of cells to HME-mediated STAT3 inhibition endowed by ectopic STAT3-C expression.
